# Promoter hypermethylation of *LGALS4* correlates with poor prognosis in patients with urothelial carcinoma

**DOI:** 10.18632/oncotarget.15865

**Published:** 2017-03-03

**Authors:** Meei-Maan Wu, Ching-Fei Li, Li-Fang Lin, Alexander Sheng-Shin Wang, Yeong-Shiau Pu, Hsiu-Hua Wang, Ai-Chung Mar, Chien-Jen Chen, Te-Chang Lee

**Affiliations:** ^1^ Department of Public Health, School of Medicine, College of Medicine, Taipei Medical University, Taipei, Taiwan; ^2^ Institute of Biomedical Sciences, Academia Sinica, Taipei, Taiwan; ^3^ Department of Urology, National Taiwan University Hospital, Taipei, Taiwan; ^4^ Genomics Research Center, Academia Sinica, Taipei, Taiwan

**Keywords:** urothelial carcinoma, galectin-4, promoter methylation, prognosis, biomarkers

## Abstract

Galectine-4 (gal-4), encoded by the *LGALS4* gene, was recently shown to exhibit a tumor suppressive effect in colorectal carcinoma and pancreatic adenocarcinoma, although how the expression of this gene is regulated remains unknown. No reports describe the significance of gal-4 in the malignant potential of urothelial tumors. Thus, we analyzed *LGALS4* methylation and gene expression and their clinical relevance and biological function in urothelial carcinoma (UC). *LGALS4* methylation was initially identified as a progression biomarker for UC patients through genome-wide DNA methylation profiling of 16 tumor samples. Bisulfite sequencing PCR and immunohistochemistry were performed to validate the promoter methylation and expression of *LGALS4*. We used quantitative methylation-specific PCR to determine the methylation levels of *LGALS4* normalized to *ACTB* in the tumor samples of 79 UC patients and compared the levels between patients with different clinicopathological characteristics. The association with survival probability was analyzed with the Kaplan-Meier method and Cox regression analysis. The ectopic expression of gal-4 in cancer cell lines was used to address its biological function in UC *in vitro*. The promoter hypermethylation of *LGALS4* (>2.51, log_10_ scale) revealed a positive correlation with high levels of both histological grade and tumor T category and with lymph node metastasis (all P≤0.001). In addition, *LGALS4* hypermethylation was an independent predictor of inferior survival in UC patients (P<0.05). The ectopic expression studies demonstrated that gal-4 suppressed urothelial cancer cell growth, migration, and invasion. Thus, *LGALS4* may function as a tumor suppressor gene in UC progression. Our findings provide evidence that methylation-mediated *LGALS4* gene repression may be involved in urothelial tumor progression.

## INTRODUCTION

Urothelial carcinoma (UC) originates from the renal pelvis, ureter, urinary bladder, and urethra. UC ranks as the 2^nd^ most common urological malignancy in many countries, including Taiwan [1–2]. Clinically, approximately 70% of these patients at first diagnosis present with treatable superficial Ta or T1 tumors; the other 25% present with more advanced stages that involve muscle invasive UC (T2 or above) or distant metastasis (T4) [3]. Approximately 60% of the treated superficial cases will recur every 5 years, and another 10-30% will progress rapidly with an unfavorable prognosis in the advanced-stage cases [3–4]. The 5-year survival rate by stage for patients diagnosed with bladder cancer dramatically declines from 70.2% at the local stage to 34.5% at the regional stage and to 5.2% at distant stages, which makes this disease a common cause of death in cancer patients (http://seer.cancer.gov/statfacts). Further understanding of the molecules involved in tumor progression and the identification of specific biomarkers for early detection before invasive disease occurs are desirable for better identification of patients at high risk of advanced-stage UC.

DNA-based biomarkers, including markers of genetic alterations and epigenetic changes, have been developed for cancer detection. DNA hypermethylation in the promoter region is the most common and best characterized epigenetic change in human malignancies [5–6]. Promoter hypermethylation alone or in combination with repressive histone modification is a mechanism that is frequently associated with transcriptional gene silencing, which contributes to tumor initiation, invasion, and metastasis in several types of cancer [7]. In UC progression, promoter hypermethylation may be more typical in invasive tumors than in superficial tumors [8]. Many biomarkers of methylation status correlate with UC, but the functional relationships with tumor development and progression or their clinical relevance to patient survival are largely unknown [9–10]. Thus, functional characterization and prognosis analysis of a given gene should be explored to determine the biological role of a methylation marker in tumor progression.

*LGALS4* encodes galactoside-binding soluble lectin 4, which is a member protein of the galectin family with 323 amino acids (galactin-4, abbreviated as gal-4). Many galectins have been identified and function in a variety of biological processes in both the intracellular and extracellular milieu [11–12]. These proteins are uniquely expressed in a tissue- or organ-dependent manner. For example, gal-4 expression is highly restricted to the luminal epithelia of the gastrointestinal tract [13–14], which is recognized as a marker of cell differentiation [15–16]. Gal-4 has been reported as a stabilizing component of adherent junctions or lipid rafts in the microvillus membrane of intestinal cells [17]. Gal-4 has also been suggested to lower the levels of cytoplasmic β-catenin and interfere with Wnt/β-catenin signaling in some malignancies [18–20]. In contrast to other galectins, such as gal-1, -2, -3, and -8, gal-4 is less frequently reported in disease predisposition. Although the functional significance of gal-4 expression correlating with biological activities has been studied as a possible cause of tumor progression and metastasis in several cancer tissues, the results are conflicting. Satelli *et al* recently demonstrated a progressive loss of gal-4 expression in colorectal cancer (CRC) and revealed that gal-4 exhibited a tumor suppressive effect in CRC cells *in vitro* [20]. The down-regulation of gal-4 expression was also observed in a more aggressive form of pancreatic cancer and was closely associated with lymph node (LN) metastasis in this cancer, which also suggests its role as a tumor suppressor in pancreatic metastasis [18–19]. In contrast, Hayashi *et al* reported that gal-4 expression was an independent predictor of LN metastasis in lung adenocarcinoma [21]. To the best of our knowledge, there are no reports describing the significance of gal-4 in the malignant potential of urothelial tumors [22].

The present study systematically identified potential methylation markers associated with UC progression using the Infinium Methylation 27K BeadChip assay. We found that the methylation level of *LGALS4* positively correlated with advanced-stage UC tumors. We further investigated the expression association and clinical relevance of the methylation status of this gene in the tumor samples of 79 UC patients. We performed *in vitro* experiments and examined biological function alterations in UC cell lines with ectopic expression of gal-4 to support its association with cancer progression. This study is the first report to provide direct evidence that methylation-mediated *LGALS4* gene repression may be involved in UC progression.

## RESULTS

### *LGALS4* CpG methylation as a candidate progression biomarker in UC patients

We performed Infinium Methylation 27K BeadChip assays to compare the methylation state of over 27,000 CpG sites associated with more than 14,000 genes in tumor DNA samples obtained from four patient groups with various stages of UC progression, including nonrecurrent-early-stage (NE), nonrecurrent-advanced-stage (NA), recurrent-early-stage (RE), and recurrent-advanced-stage (RA) UC groups. Each group consisted of 4 patient samples for pooled DNA (Supplementary Table 1). The results of an unsupervised clustering analysis utilizing all of the informative CpG sites revealed that the DNA methylation profile in advanced-stage UC was distinct from that in early-stage UC (data not shown). Comparisons between these two groups demonstrated that 69 CpG sites showed an absolute beta difference (Δβ) value meeting a 0.4 threshold. We then refit a Δβ-distributed heatmap to the UC samples including these 69 CpG sites. Supplementary Figure 1 shows that we identified 27 hypermethylated CpG sites associated with 23 genes and 42 hypomethylated CpG sites associated with 40 genes that exhibited Δβ values between the early- and advanced-stage UC groups. *LGALS4* was included in the list of hypermethylated genes with a Δβ equal to 0.4115 (Supplementary Table 2).

**Figure 1:**
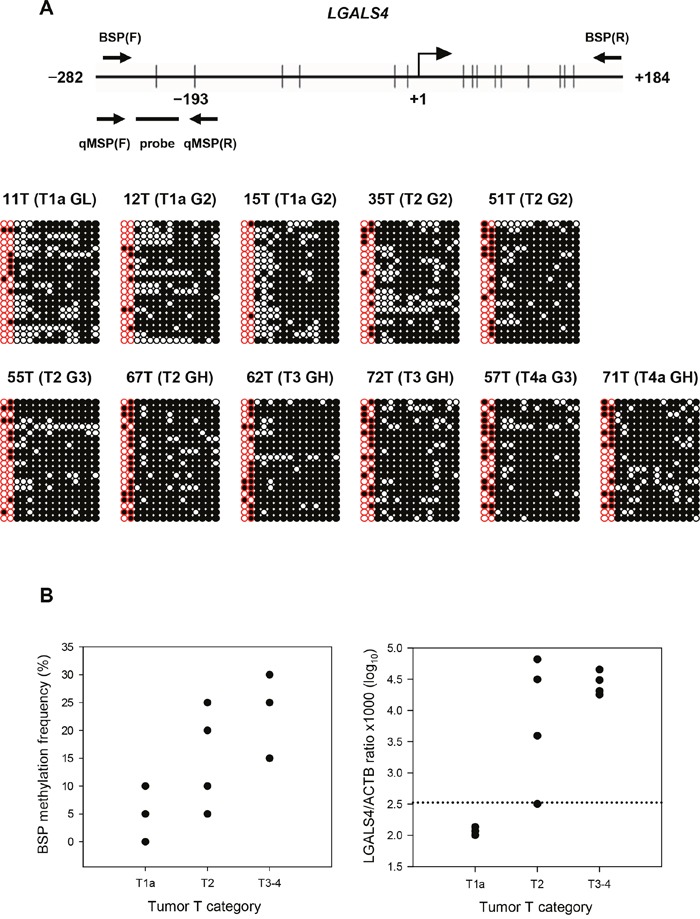
Results of bisulfite sequencing PCR (BSP) and a comparison of *LGALS4* CpG methylation levels with the results of quantitative MSP (qMSP) assay using 11 samples of UC **(A)** Distribution of 15 CpG sites within the promoter of *LGALS4* spanning -252 to +184 nts. for the BSP method. +1: transcription start site. The first two CpG dinucleotides were analyzed in qMSP assay including the significant loci located at -193, as identified by the Infinium Methylation 27K BeadChip assay. The cloning results of the BSP for 11 UC samples are shown. Each row illustrates a clone, and column a single CpG site. Open and filled circles represent methylated and unmethylated CpG, respectively. The T category of the AJCC system and histological grade, G, are indicated in the parenthesis for each sample. H: high. **(B)** Figures show a comparison between the BSP (*left*) and qMSP (*right*) data for the first two CpG sites within the *LGALS4* gene promoter in UC samples by tumor T category.

We developed a quantitative methylation-specific PCR (qMSP) assay for a clinical association study of 79 UC patients (Table 1) to assess the role of *LGALS4* in UC progression. We designed primer pairs and a probe based on the initial finding of a locus located at -193 nucleotides (nts) in relation to the transcription start site (+1 nt), as identified by the Infinium methylation assay, to evaluate the methylation status of two CpG dinucleotides in the promoter of the *LGALS4* gene (Figure 1A). We first performed a bisulfite sequencing PCR (BSP) assay using 11 UC DNA samples and compared the results to those of the qMSP method to determine whether the qMSP method reflected the methylation pattern of the *LGALS4* promoter. Figure 1A shows the cloning results. The plot illustrates that the first two CpG sites were highly methylated in advanced-stage UC compared to early-stage UC when analyzed using BSP, with a median frequency of 20.0% in the T3 or T4 tumors, 15% in the T2 tumors, and 5% in the T1a tumors (Figure 1B, *left*). Analysis using the qMSP method revealed a consistent result (Figure 1B, *right*). The median methylation level was 4.40, 4.04, and 2.07 in a log_10_ scale for T3 or T4, T2, and T1a, respectively.

**Table 1:** Demographic and clinicopathological features of 79 patients with urothelial carcinoma

Variable	Value*
Age, year (IQR)	66.6 (59.5-73.7)
Gender	
Male	51 (64.6)
Female	28 (35.4)
Location of tumor	
Urinary bladder	73 (93.6)
Ureter	2 (2.6)
Renal pelvis	3 (3.8)
Missing	1
Histological grade	
2	12 (16.0)
Low	12 (16.0)
3	15 (20.0)
High	36 (48.0)
Missing	4
Tumor T category	
Ta-1	28 (35.9)
T2	23 (29.5)
T3	16 (20.5)
T4	11 (14.1)
Missing	1
Lymph node metastasis	
0	58 (75.3)
1	8 (10.4)
2	11 (14.3)
Missing	2
Distant metastasis	
Absent	71 (92.2)
Present	6 (7.8)
Missing	2
Vital status during follow-up	
Alive	53 (67.1)
UC-specific mortality	19 (24.1)
Non-UC mortality	7 (8.9)

* Age is presented as median (IQR, interquartile range). The other variables are presented as number (percentage).

### Immunohistochemistry of gal-4 expression in UC tissue sections

**Figure 2:**
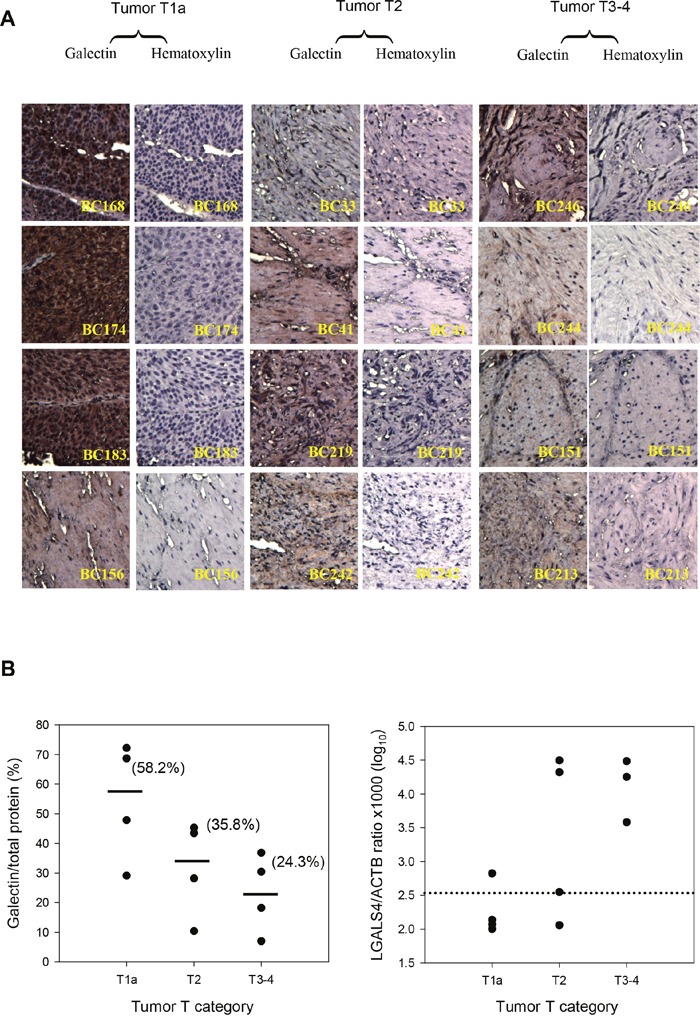
*LGALS4* protein expression (gal-4) and methylation levels in UC samples by tumor T-category **(A)** Results of immunohistochemical expression of gal-4 (x20 magnification) in tissue sections. **(B)** Histoscores of gal-4 expression levels in UC tissue section (*left*). Solid lines and the number in parenthesis represent the median value within a group of T-categories. The distribution of *LGALS4* methylation levels in UC samples (*right*). The data of qMSP assay for the BC213 sample is missing. The dotted line indicates the cut-off value, 2.51 in a log_10_ scale, which was used to define a sample as high (>2.51) or low methylation status.

We next performed immunohistochemical (IHC) analysis to evaluate gal-4 protein expression in 12 samples of UC in relation to the tumor T category because of the potential biological relevance of *LGALS4* expression in cancer progression. Figure 2A shows that gal-4 was highly expressed in the low-T-category UC and that the high-T-category UC samples expressed a reduced level of gal-4. The median histoscores of the tumor tissues were 58.2%, 35.8%, and 24.3% in the tissue sections of the T1a, T2, and T3 or T4 UC groups, respectively, which indicated a decreasing level of gal-4 expression in parallel with UC tumor progression (Figure 2B, *left*). The qMSP analysis of these samples revealed that the proportion of the samples that exhibited hypermethylation (>2.51, log_10_ scale) was 25%, 75% and 100% of the samples in the T1a, T2, and T3 or T4 categories, respectively (Figure 2B, *right*). The proportion of the samples that exhibited hypermethylation, determined using the qMSP analysis, was consistent with the expression levels of gal-4 from the IHC analyses.

### *LGALS4* CpG methylation, clinicopathological characteristics and prognosis

**Figure 3:**
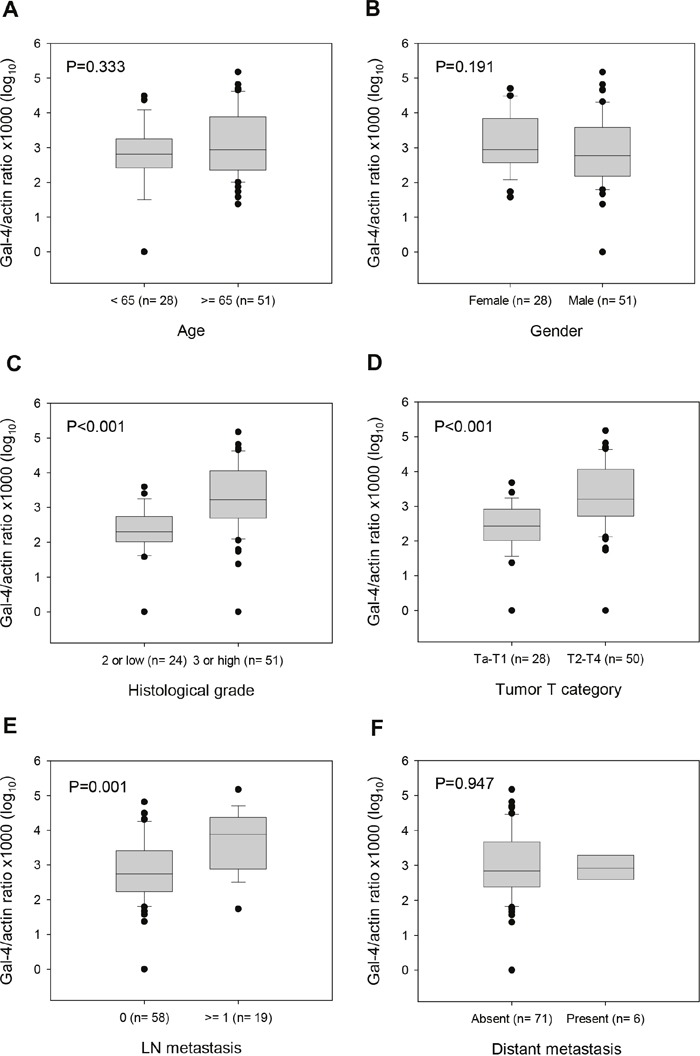
Box-plot describing *LGALS4* methylation levels in UC patients by clinicopathological factors **(A)** Patients <65 years vs. ≥65 years of age at surgery. **(B)** Patients female vs. male. **(C)** Specimens displaying low vs. high histological grade. **(D)** Specimens displaying low vs. high tumor T category. **(E)** Specimens displaying absent vs. present LN metastasis. **(F)** Specimens displaying absent vs. present distant metastasis. The methylation levels were expressed in log_10_ scales. Black circles represent the values outside the box range. The P values were derived from Mann-Whitney *U* test.

We analyzed the correlation between *LGALS4* methylation and various clinicopathological factors, including age, gender, histological grade, tumor T category, LN metastasis and distant metastasis, to evaluate the clinicopathological significance of the *LGALS4* methylation status in UC. Figure 3 shows the distribution of the *LGALS4* methylation levels in the tissue samples from 79 UC patients according to various clinicopathological factors. Analysis using the Mann-Whitney *U* test indicated significantly higher methylation levels in the groups with a high grade (Figure 3C) and a high T category (Figure 3D) or the presence of LN metastasis (Figure 3E) (all P≤0.001). However, there were no substantial differences between the groups with regard to age, gender, or distant metastasis (Figure 3A, 3B, and 3F, respectively).

**Figure 4:**
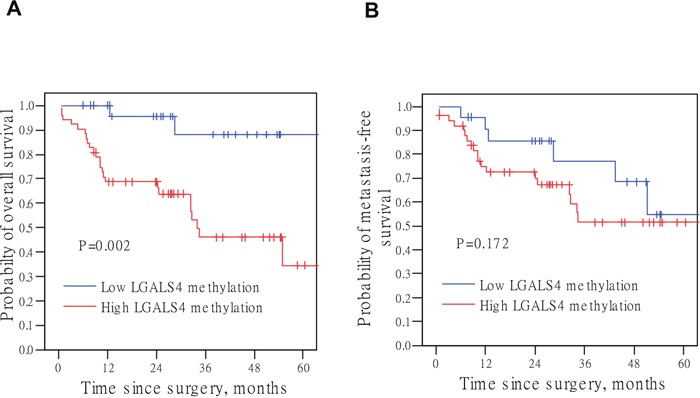
Kaplan-Meier curves for survival analysis with log-rank P values **(A)** Overall survival probability. **(B)** Metastasis-free survival probability. *LGALS4* methylation level of 2.51 in a log_10_ scale was the cut-off point to classify samples into low or high methylation group. This value approximately represents the lowest tertiary value of the distribution among the total patient subjects. The results of an initial Kaplan-Meier analysis revealed a similar probability curve for the two upper tertiles (intermediate and high methylation levels) as shown in Supplementary Figure 2A and 2B. The P values were derived from the log-rank test.

We next examined the association of *LGALS4* methylation with overall survival or (distant) metastasis-free survival. The Kaplan-Meier analyses revealed that the overall survival rate was significantly lower in the high *LGALS4* methylation group than in the low methylation group (P=0.002, Figure 4A). Although not statistically significant (P=0.172), patients with high *LGALS4* methylation levels showed a trend towards a lower probability of metastasis-free survival (Figure 4B). The UC-specific survival analysis also revealed a result similar to that of the overall survival analysis (P=0.012, Supplementary Figure 2C). Cox proportional hazard regression analysis revealed that the UC group with high *LGALS4* methylation levels had a 7.36-fold increased risk of mortality compared to the low methylation group (Table 2, Model I). The multivariate Cox analyses further demonstrated that a high methylation level remained a significant prognostic factor for a decreased survival rate, independent of the histological grade (P=0.033), tumor T category (P=0.044), or LN metastasis (P=0.018) (Table 2, Model II, III, and IV, respectively). The multivariate Cox regression analysis revealed that LN metastasis and a high *LGALS4* methylation level were the two most important predictors for a shortened survival in UC patients (Table 2, Model V, both P=0.061).

**Table 2:** Univariate and multivariate Cox hazard analysis of overall survival in patients with urothelial carcinoma

Variable	Univariate analysis			Multivariate analysis											
	Model I			Model II			Model III			Model IV			Model V		
	HR*	95% CI*	P	HR	95% CI	P	HR	95% CI	P	HR	95% CI	P	HR	95% CI	P
Age, one year increment	1.01	(0.98-1.05)	0.503	1.02	(0.98-1.05)	0.340	1.02	(0.98-1.06)	0.310	1.02	(0.99-1.06)	0.212	1.02	(0.99-1.06)	0.239
Male vs. female	0.66	(0.31-1.44)	0.299	0.79	(0.36-1.76)	0.568	0.84	(0.38-1.86)	0.673	0.77	(0.35-1.71)	0.516	0.79	(0.35-1.78)	0.566
Grade 3 or high vs. 2 or low	4.36	(1.31-14.56)	0.017	2.13	(0.59-7.72)	0.252							0.98	(0.22-4.39)	0.975
T2-4 vs. Ta-T1 stage	7.12	(1.68-30.25)	0.008				3.94	(0.88-17.57)	0.072				2.80	(0.51-15.51)	0.239
LN metastasis, present	3.20	(1.45-7.08)	0.004							2.40	(1.05-5.48)	0.038	2.27	(0.96-5.35)	0.061
*LGALS4* methylation**															
High vs. low levels	7.36	(1.74-31.19)	0.007	5.44	(1.15-25.70)	0.033	4.68	(1.04-21.04)	0.044	5.95	(1.37-25.91)	0.018	4.51	(0.94-21.75)	0.061

* HR: hazard ratio, CI: confidence interval.

** The lowest tertiary cut-off point of 2.51 (log_10_ scale) was used to classify samples into low or high methylation group.

### Epigenetic mode is involved in the decreased *LGALS4* expression in urothelial cells

**Figure 5:**
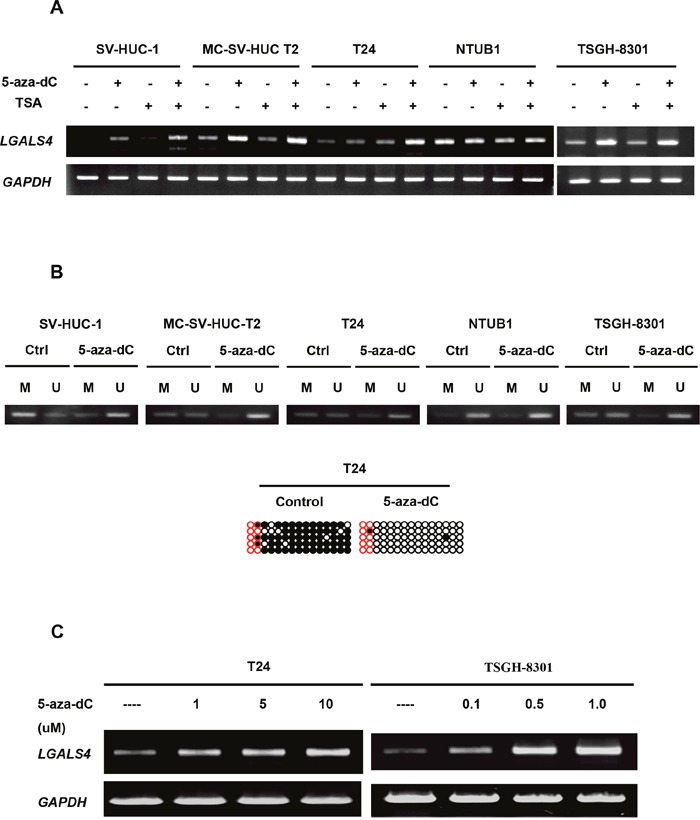
Transcripts levels of *LGALS4* in five human urothelial cell lines before and after epigenetic modifier treatment **(A)** *LGALS4* transcripts as well as *GAPDH* transcripts (internal control) were analyzed by RT-PCR. 5-aza-dC: a DNA methyltransferase inhibitor, 1 μM treatment during cell culturing. TSA: a histone deacetylase inhibitor, 0.1 μM treatment in cell culture. **(B)** Methylation status of the cell lines before and after modifier treatments by MS-PCR and by BSP method (T24 cells only). **(C)** A dose-response analysis of *LGALS4* gene expression after 5-aza-dC treatments in T24 or TSGH-8301 cells.

High *LGALS4* methylation levels were closely associated with UC progression. Therefore, we conducted *in vitro* studies using human urothelial cell lines to evaluate whether epigenetic silencing contributed to the decrease in *LGALS4* expression. We performed RT-PCR to assess the treatment effect of 5-aza-dC and/or trichostatin A (TSA) in five urothelial cell lines. As shown in Figure 5A, the SV-HUC-1, T24 and TSGH-8301 cell lines expressed relatively lower levels of *LGALS4* transcripts, while the MC-SV-HUC T2 and NTUB-1 cell lines expressed higher levels of *LGALS4* transcripts before either treatment. Treatment with 5-aza-dC and/or TSA significantly increased the *LGALS4* transcripts in all of the cell lines, especially in the SV-HUC-1, T24 and TSGH-8301 cells. The methylation status changes of those cell lines before and after the modifier treatments were confirmed by MS-PCR for all 5 of the cell lines and by BSP for the T24 cell line (Figure 5B). A dose-response analysis of the gene transcripts after 5-aza-dC treatment in the T24 or TSGH-8301 cell lines further confirmed the role of promoter hypermethylation in the decreased *LGALS4* expression in T24 and TSGH-8301 cells (Figure 5C). Therefore, we selected the T24 and TSGH-8301 cells with strong repression of *LGALS4* expression for subsequent analyses.

### Effects of gal-4 overexpression in urothelial cancer cells *in vitro*

**Figure 6:**
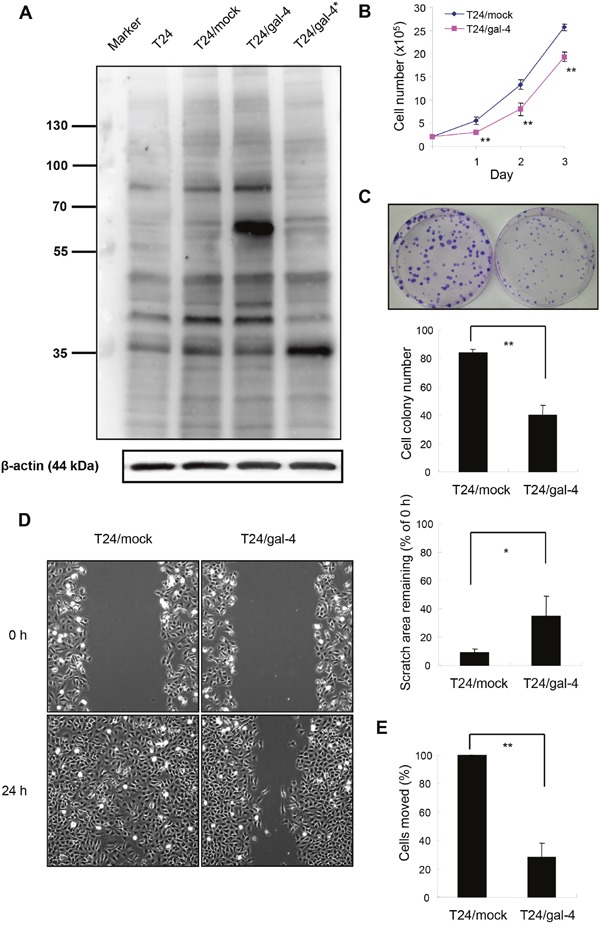
Effects of *LGALS4* protein expression (gal-4) in T24 transfectants The cells were transfected with either the pCMV6-AC-GFP/gal-4 (T24/gal-4 cell line) or empty vector (T24/mock cell line) as a control. **(A)** Ectopic expression of gal-4 in T24 transfectants. Proteins from the whole extracts of T24/mock or T24/gal-4 cells were analyzed by Western analysis for the detection of gal-4 or β-actin (as a loading control). The lane beside the markers was run with the extracts of T24 cells as a contrast. T24/gal-4* was T24 transfectants containing the pIRES-EGFP/gal-4 vector, which showed gal-4 expression (36 kD) only as a contrast. **(B)** Cell proliferation of T24/mock and T24/gal-4 cells, as determined in cell viability assays using trypan blue exclusion method. Bars represent the mean± SEM of three independent experiments performed in duplicate. ** P<0.01. **(C)** Colony formation of T24/mock and T24/gal-4 cells. One hundred cells each were initially seeded in 10-mm dish and cultured for 10 days. The cell colonies were stained and counted by the Giemsa method. Columns and bars represent the mean± SEM of four independent experiments performed in triplicate. ** P<0.01. **(D)** Migration capability of T24/mock and T24/gal-4, as measured in wound healing scratch assay. Representative photographs at time points 0 and 24 h after a scratch treatment. Histographic presentation of the data analyzed from the photographs taken at 0 h and 24 h after the scratch. Columns and bars represent the mean± SEM derived from three separate experiments. ** P<0.01. **(E)** Invasion capability of T24/mock and T24/gal-4, as assessed by moving over gelatin-coated transwell chambers. Columns and bars represent the mean± SEM derived from three independent experiments performed in duplicate or triplicate. ** P<0.01.

We first constructed T24 cancer cells that overexpressed gal-4 by introducing a vector containing *LGALS4* cDNA (T24/gal-4 cell line) and then assessed the possible effects of gal-4 expression on the proliferation, migration and invasion of the cell line to examine whether *LGALS4* gene products influence the behavior of T24 cells. A vector without the insert was transfected into T24 cells as a control (T24/mock cell line). The band identified at approximately 61 kDa by Western blot analysis corresponded to the mass of a fusion protein of GFP (25 kDa) and gal-4 (36 kD). This band was observed in the T24/gal-4 cells extract, whereas the T24/mock cell extract exhibited no detectable expression of the fusion protein (Figure 6A). These results demonstrated that gal-4 was ectopically overexpressed in the T24/gal-4 cell line. Cell viability analysis of these two cell lines indicated that T24/gal-4 cells exhibited a statistically significant decrease in cell proliferation compared to the T24/mock controls (Figure 6B). There was also a significant decrease in the colony formation of T24 cells expressing gal-4 compared to the control cells without gal-4 (Figure 6C). Next, the T24/gal-4 and T24/mock cell lines were used to examine whether ectopically expressed gal-4 influenced the migratory and invasive properties of the T24 cells using the wound healing assay and the transwell chamber assay, respectively. Figure 6D shows that T24/gal-4 cells displayed a significant decrease (50%) in their migration ability after 24 h compared to the T24/mock cells (P<0.01). Cells expressing gal-4 were also less invasive over filters than were cells without the gal-4 insert, with an approximate reduction of 70% (Figure 6E). These results indicated that gal-4 restricted or delayed the proliferation, migration, and invasive capabilities of T24 cells following gal-4 overexpression. Importantly, we repeated the *in vitro* experiments using TSGH-8301 cells as the transfectants and obtained similar results regarding the inhibitory effects of gal-4 overexpression on cell proliferation, migration and invasion, as shown in Supplementary Figure 3.

## DISCUSSION

Many studies have reported that methylation markers can differentiate subgroups of UC. However, most of these methylation markers were associated with recurrence and relatively few studies on advanced stages have been reported. Patients with advanced stages such as muscle-invasive and metastatic tumors comprised 30~40% of total UC cases, and their 5-year survival probability is less than one third. In our study, advanced-stage UC correlated with a high level of promoter methylation in *LGALS4* compared with early-stage UC. Higher methylation levels also correlated with a progressive decrease in protein expression and significantly predicted an inferior survival for UC patients. Studies of ectopic expression in urothelial cell lines further established that this gene product affects several carcinogenic phenotypes, which suggests a biological role in carcinogenesis. These findings may provide a treatment option for advanced-stage patients because DNA methylation is reversible.

Down-regulation of gal-4 expression has been reported in colon adenoma/carcinoma and pancreatic adenocarcinoma [18–20]. However, the mechanisms regulating expression of the gal-4 gene (*LGALS4*) have not yet been elucidated. We demonstrated that decreased expression and increased promoter methylation of this gene were associated with a trend toward a more advanced stage of UC. A previous genomic analysis by Selamat *et al* found hypermethylation of *LGALS4 in* lung adenocarcinoma in smokers [23], and their studies further indicated an inverse association between promoter hypermethylation and expression of *LGALS4* transcripts. Collectively, these results together with our study suggest that DNA methylation is a potential regulator of *LGALS4* expression. However, the correlation between promoter hypermethylation and protein expression in our study may have been a chance finding because of the limited number of samples in our analyses. Therefore, we performed *in vitro* experiments using demethylation treatment with 5-aza-dC alone or in combination with TSA, and the results revealed significant restoration of gal-4 expression. These findings are likely the results of promoter hypermethylation alone or in combination with a repressive histone modification. Thus, the activities of DNA methyl transferase and histone deacetylases in UC tumors should be examined to justify the causal relationship between hypermethylation and the reduced expression of the *LGALS4* gene.

Gal-4 is normally not detected in healthy urothelia, although it is present at varying levels in urothelial tumors [24]. IHC analyses of tumor samples by Langbein *et al* indicated a trend towards more intense reactivity in superficial tumors, but expression was most frequently negative in high-grade, high-stage (T-category) carcinomas [25]. The function of gal-4 in the development and progression of UC remains unclear, although a similar pattern of expression was observed in our study. The protein levels of gal-4 were low in T3 or T4 UC, intermediate in T2 UC, and high in T1 or Ta UC. We also compared cell proliferation, migration, and invasion in cell lines with forced expression versus endogenous expression of gal-4, and the results provided direct evidence that gal-4 expression inhibited the malignant properties of urothelial cancer cells *in vitro*. However, the mechanism for how gal-4 inhibits the malignant properties of UC remains to be explored. Gal-4 may contribute to the inhibition of migratory/invasive behaviors by stabilizing the destruction complex, which decreases the expression of target genes in the Wnt/β-catenin signaling pathway, as shown for CRC and pancreatic cancer [19–20]. Gal-4 may also modulate the adhesion of tumor cells to vascular cells, which mediates venous invasion and metastasis in several tumor types [21, 26]. However, these latter studies considered gal-4 to be a risk factor for the acquisition of tumor malignancy; an opposite role as suggested would hardly explain the repression of gal-4 in the malignancy-associated process of UC cells in our study.

Complex interactions are required between the molecular determinants of progression and invasion leading to the detachment of epithelial cells from primary tumors [27]. Aberrant activation of the epithelial-to-mesenchymal transition (EMT), characterized by the loss of homotypic adhesion and basal-apical polarity that triggers malignant progression, is a plausible mechanism for the formation of malignant UC [28]. Notably, the overexpression of integrin-linked kinase was recently identified in UC in a genome-wide profiling analysis [29], and its importance in the regulation of UC invasion was reported [30]. Integrin molecules are transmembrane glycoproteins, the altered distributions of which are frequently observed in the invasion of malignant tumors, including UC [31]. The expression of certain integrins is closely associated with the progression to invasive carcinoma or increased metastatic potential [32]. Therefore, further studies investigating the interaction of gal-4 with integrins in mediating EMT are warranted and are currently underway in our research group.

In conclusion, we identified a high level of *LGALS4* promoter methylation in high-grade and high-T-category UC and observed an association with reduced gal-4 expression in tumor tissues. The promoter hypermethylation of LGALS4 might have potential use in the diagnostic panel as a differential detection marker for advanced-stage UC subtypes. Our clinical analysis further revealed an association with inferior survival independent of histological grade and tumor T category, which suggests the predictive value of *LGALS4* promoter methylation as a prognostic biomarker in patients with advanced UC. Furthermore, the results of our gal-4 overexpression analyses suggest that reactivation may suppress UC cell migration and invasion. Therefore, the *LGALS4* promoter might be a potential target site for advanced UC in cancer therapies.

## MATERIALS AND METHODS

### Patients and sample collection

This study included 84 UC samples from patients who were surgically treated at the National Taiwan University Hospital (NTUH) from April 2002 to November 2008. The surgical samples were obtained by trans-urethral resection for the non-muscle-invasive patients (Ta or T1) or by radical cystectomy for the muscle-invasive patients (T2 or beyond). A portion of the resected specimens was fixed in 10% formalin and embedded in paraffin (FFPE). Another portion of the specimens was snap-frozen and stored in liquid nitrogen immediately after surgery until further use for DNA extraction. More than 92% of the tumor samples were removed from the location of the urinary bladder, and the remaining were from the renal pelvis or ureter. All of the samples were histopathologically confirmed as UC. The patient demographic data, including age, gender and clinicopathological characteristics, were retrieved from the hospital records.

We prepared a discovery set using the snap-frozen tissues obtained from the 16 patients for DNA extraction to examine the differential methylation profiles associated with UC progression. The patient samples of this set were randomly selected from the total patients based on a combination of recurrent status and overall cancer stage and were grouped into four groups, including nonrecurrent-early-stage (NE), nonrecurrent-advanced-stage (NA), recurrent-early-stage (RE), and recurrent-advanced-stage (RA), with each group consisting of 4 patient samples for pooled DNA. The AJCC TNM category and the overall cancer staging of the 16 patients are summarized in Supplementary Table 1. The frozen tissues from the 79 UC patients were included for the qMSP analysis. Table 1 summarizes the demographic and clinicopathological features of the 79 UC patients. Among the 79 patients, 11 and 11 samples with no LN or distant metastasis were also used for the BSP and IHC analyses, respectively. Written informed consent was obtained from all of the patient subjects. Both the Institutional Review Boards of NTUH and Academia Sinica, Taiwan, approved this study.

### DNA extraction

The UC frozen tissues were lysed with 1.0 mL DNAzol reagent (Molecular Research Center, Cincinnati, OH, USA) and 200 μg/mL proteinase K (Sigma-Aldrich, St. Louis, MO, USA) by gentle pipetting. A 0.5-mL aliquot of chloroform was added to the cell lysate. This mixture was shaken vigorously and was then centrifuged at 12,000 rpm for 5 min. After centrifugation, the supernatant and the DNA pellet were collected. The DNA was dissolved in 50 μL of 8 mM NaOH and was quantified with a NanoDrop ND-1000 spectrophotometer (Thermo Fisher Scientific, Waltham, MA, USA) for the determination of double-stranded DNA (For details, see the online Supplementary MATERIALS AND METHODS).

### Genome-wide DNA methylation profiling

The genomic DNA obtained from each sample of the similar UC progression groups was pooled in equal amounts (Supplementary Table 1), and 0.5 μg of the pooled DNA was modified with sodium bisulfite using the EZ DNA Methylation-Gold Kit (Zymo Research, Orange, CA, USA) according to the manufacturer's protocol. The methylation profiles of the modified DNA were obtained using the Infinium Methylation 27K BeadChip assay (Illumina, San Diego, CA, USA), and the CpG loci were measured using Illumina BeadStudio software (Genetech Biotech, Taipei, Taiwan). The beta (β) values were calculated by subtracting the background using the negative controls on the array and taking the ratio of the methylated signal intensity to the sum of both the methylated and unmethylated signals plus a constant of 100. A β value of 0 to 1.0 was reported for particular CpG loci and represented a percent methylation of 0% to 100%, respectively. Two measurements with a detection level of P>0.05 were marked as missing and were subsequently excluded from the analysis, which left 27,576 informative loci for the analysis.

### Bisulfite sequencing PCR (BSP)

Bisulfite genomic sequencing was used to examine the methylation status of the CpG sites spanning the region of -252 to +184 within the *LGALS4* promoter. The DNA extraction and the bisulfite modification were performed as previously described for DNA methylation profiling. The primers flanking the region were 5′-GTTTTGATAAGGTTTTGGT-3′ (forward) and 5′-CCCCCAAAATCAAAATAAAA-3′ (reverse). In a final volume of 25 μL, 25 ng of bisulfite-modified DNA was mixed with 2.5 μL of 10x PCR buffer, 1.25 μL of AmpliTag Gold, 500 nM of each primer, 200 nM of each dNTP, and 5 mM of MgCl_2_. The PCR reaction was performed at 95°C for 10 min, followed by 30 cycles of 95°C for 15 sec, 55°C 15 sec, 60°C for 1 min, and a 6-min extension at 72°C.

The PCR products were purified and subcloned into the pCR4-TOPO vector using the TOPO TA Cloning kit (Life Technologies, Carlsbad, CA, USA) followed by transformation into *E. coli* competent cells (Life Technologies). Twenty clones per sample were expanded overnight, and the plasmid DNA was extracted using the QIAprep Spin Miniprep kit (Qiagen, Valencia, CA, USA). The purified plasmid DNA was subjected to a sequence analysis at the DNA Analysis Facility at the Institute of Biomedical Sciences, Academia Sinica, Taiwan.

### Quantitative methylation-specific PCR (qMSP)

We performed a DNA methylation analysis using the fluorescence-based, real-time PCR assay, MethyLight, as previously described [33]. The DNA extraction and the bisulfite modification were performed as previously described for DNA methylation profiling. The sequences of the paired primers and the TaqMan probe used to amplify and detect fully methylated *LGALS4* in the promoter region were 5′-GTTAATAGAAGTTTGGGTAGGGT-3′ (forward), 5′-CTAAATCCCCTCCCCTACG-3′ (reverse), and 6FAM5′-AGGGTCGAAGTTTATGAGTATTTTTTTTT-3′TAMRA (probe).

An unmethylated sequence of the housekeeping gene β-actin (*ACTB*) was amplified and included in each assay as a DNA loading control. The primer and probe sequences used to amplify *ACTB* were designed according to the sequences published by Eads *et al* [34]. We used *in vitro* methylation to generate a standard calibration curve and quantify the amount of methylated alleles of *LGALS4* in each sample, and the concentration of an unknown sample was derived from the curve. Leukocyte DNA from a healthy individual was methylated with SssI methyltransferase (New England Biolabs, Ipswich, MA, USA), and the methylation status of the DNA was examined for completeness with the BSP method as shown in Supplementary Figure 4. We then prepared serial dilutions (400~0.4 ng) of the completely methylated DNA to construct the standard curve, which was included in each plate for the qMSP quantification.

The amplification reactions of the qMSP assays were performed in 96-well plates sealed with an adhesive film. The plates were read using a Roche LightCycler 480 Detector System (Roche Diagnostics, Foster City, CA, USA). Each PCR reaction was performed in a final volume of 20 μL containing 2 μL of 10x PCR buffer; 0.5 U of Gold Taq DNA polymerase (PE Applied Biosystems, Foster City, CA), 600 nM each of forward and reverse primers, 200 nM of TaqMan probe, 200 μM each of dATP, dCTP, dGTP, and dUTP, 3.5 (*LGALS4*) or 5.5 (*ACTB*) mM of MgCl_2_, and 3 μL of bisulfite-modified DNA. The thermocycling conditions consisted of 10 min at 95°C followed by 50 cycles of 15 sec at 95°C and 1 min at 60°C. All of the samples were analyzed in duplicate. The level of methylated *LGALS4* DNA in a particular sample was determined as a ratio of the derived concentration for the *LGALS4* PCR products to the *ACTB* PCR products and was multiplied by 1,000. All of the values of the methylation levels in this study are presented in base 10 logarithmic scales.

### Immunohistochemistry (IHC)

The protein expression of *LGALS4* in the FFPE specimens was determined using an IHC assay based on the avidin-biotin-peroxidase method (see the online Supplementary MATERIALS AND METHODS).

### Cell lines

Five human urothelial cell lines, SV-HUC-1, MC-SV-HUC T2, T24, NTUB1, and TSGH-8301, were used. The SV-HUC-1 (CRL9520) immortalized cell line and the MC-SV-HUC T2 (CRL9519) and T24 carcinoma cell lines were purchased from the American Type Culture Collection (Manassas, VA, USA). The former 2 cell lines were used in our previous studies and were validated and maintained as previously described [35–36]. The NTUB1 and TSGH-8301 carcinoma cell lines were provided by Dr. Yeong-Shiau Pu (an investigator in the current study). These 2 cell lines and the T24 cells were cultured under the conditions previously described [37–40].

### RNA extraction and reverse transcriptase-PCR (RT-PCR)

Total RNA from the cultured cells was extracted using the TRI reagent (Molecular Research Center), according to the manufacturer's protocol (see the online Supplementary MATERIALS AND METHODS).

### 5-aza-2′-deoxycytidine (5-aza-dC) and trichostatin A (TSA) treatment

We examined the gene expression of *LGALS4* in several human urothelial cell lines exposed to 5-aza-dC (Sigma-Aldrich), a DNA methyltransferase inhibitor, and/or TSA (Sigma-Aldrich), a histone deacetylase inhibitor, to examine the potential involvement of epigenetic silencing in *LGALS4* repression (see the online Supplementary MATERIALS AND METHODS).

### Methylation-specific PCR (MS-PCR)

The DNA extraction and the bisulfite modification were performed as previously described for DNA methylation profiling. The primers used for detecting methylated and unmethylated CpG sites of -227 and -193 nts in the promoter of the LGALS4 were pairs of 5′-GATAAGGTTTTGGTAGGGTCGAA-3′ (forward)/5′-CTAAATCCCCTCCCCTACG-3′ (reverse) and 5′-GATAA GGTTTTGGTAGGGTTGAA-3′ (forward)/5′-CTAAAT CCCCTCCCCTACA-3′ (reverse), respectively. The MS-PCR reaction was carried out using the i-StarTag GH DNA polymerase purchased from the iNtRON Biotechnology (Sungnam, Kyungki-Do, Korea). An aliquot of 10 μl of each PCR product was analyzed by agarose gel electrophoresis.

### Construction of UC cell lines ectopically expressing gal-4

We selected the T24 and TSGH-8301 cell line with strong repression of *LGALS4* expression for the *in vitro* experiments. Full-length *LGALS4* cDNA in the plasmid was a generous gift from Dr. Fu-Tong Liu (Academia Sinica, Taipei, Taiwan) and was amplified by RT-PCR with the sense primer 5′-GCG ATC GAA TGG CCT ATG TCC CCG CAC C-3′ and the antisense primer 5′-TCG CGT GAT CTG GAC ATA GGA CAA GGT G-3′, containing SgfI/MluI restriction sites, respectively, for insertion. The PCR product was digested and subcloned into the pCMV6-AC-GFP vector (Origene, Rockville, MD, USA) under the control of a CMV promoter. The construct was propagated in *E. coli* (Life Technologies) and was purified using a spin-column plasmid isolation kit from Qiagen. The *LGALS4* cDNA sequence was verified using a direct sequencing analysis before subsequent transfection.

Transfection of T24 cells with the plasmid expressing the *LGALS4* protein gal-4 (pCMV6-AC-GFP/gal-4), abbreviated as the T24/gal-4 cell line, was performed using the FuGENE® HD Transfection Reagent (Promega, Madison, WI, USA), according to the manufacturer's instructions. A control cell line was also constructed with the introduction of the plasmid without the gal-4 insert (T24/mock cell line). Both cell lines were selected by continuous culturing for 2~3 passages in the presence of 500 μg/mL geneticin G418 sulfate (Gibco-Life Technologies, Grand Island, NY, USA). Transfection of TSGH-8301 cells with plasmids carrying (TSGH-8301/gal-4 cell line) or not carrying the gal-4 insert (TSGH-8301/mock cell line) was carried out as described above. After 48 h of transfection, the TSGH-8301 transfectants were harvested for subsequent *in vitro* experiments.

### Western blotting analysis

Western blot analysis was performed as previously described [35–36]. Briefly, the cells were harvested and lysed. After quantitation, equal amounts of cell lysates were separated by SDS-PAGE on a 10% polyacrylamide gel, and the proteins were transferred to PVDF membranes (GE Healthcare, Munich, Germany). The membranes were blocked for 60 min at room temperature, washed and incubated overnight with primary antibodies against gal-4 (for T24 transfectants, Santa Cruz Biotechnology, Santa Cruz, CA, USA), tGFP (for the TSGH-8301 transfectants, Origene, Rockville, MD, USA) and β-actin (Abcam, Cambridge, MA, USA) as a loading control (see the online Supplementary MATERIALS AND METHODS).

### *In vitro* studies: proliferation, migration and invasion assays

Cell proliferation was determined by trypan blue exclusion (Gibco-Life Technologies). T24 and TSGH-8301cells, with pCMV6-AC-GFP/gal-4 or an empty vector, were seeded into 6-cm plates at a density of 200,000 cells per well. The cells were cultured in complete media containing 10% FBS. A cell suspension was mixed with 0.4% trypan blue (1:1) and was examined under a microscope to determine the number of viable cells. The proliferation rate was counted on days 0, 1, 2, and 3. The experiments were performed in duplicate three independent times. Colony formation assays were used to compare the ability of two T24 transfectants to grow into a colony. One hundred transfected cells were plated in a 100-mm dish and were grown for 10 days. The colonies generated were then fixed, stained and counted using the Giemsa method. The experiments were performed three independent times.

A wound healing scratch assay was used to assess the migratory capability of the T24 and TSGH-8301 cells bearing pCMV6-AC-GFP/gal-4 or the empty vector. The cells were grown to 90% confluency in 6-well plates, and a straight line scratch was made with a sterile 200-μL pipette tip in all of the wells. The scratch resulted in a cell-free gap (wound) on the cell monolayer. The width of the wound was reduced at different time intervals. Photographs of the gap width were taken under an inverted microscope connected with a Leica DMI 6000B camera at 0 h and 24 h after the scratch. Measurements were taken at 3 predefined sites along the scratch, and the average measurement was used as the mean gap width for each scratch assay. The gap area at 0 h was set to 100%, and the percentage of the area at subsequent time points relative to time 0 h was calculated as follows: (mean gap area at 0 h – mean gap area at 24 h)/ mean gap area at 0 h.

The invasion capability of the T24 and TSGH-8301 transfectants was determined using a Transwell Permeable Support assay (Corning, Lowell, MA, USA), according to the manufacturer's instructions. Briefly, an upper chamber containing a polycarbonate filter (8-μm pore size) was coated with 1% gelatin in 100 μL of 1% FBS growth medium and the lower chamber contained 600 μL of 5% FBS growth medium. A total of 5,000 T24 or TSGH cells were plated on the gelatin layer in the upper chamber and were allowed to move toward the growth media in the lower chamber overnight. The non-invasive cells inside the upper chamber were removed, and the invasive cells outside of the filter were fixed with methanol, stained with DAPI, and counted under a microscope.

### Statistical analysis

The distribution of the *LGALS4* methylation levels of the UC samples in relation to clinicopathological factors was graphed using box plots in SigmaPlot for Windows v.8.02 (SPSS Inc, Chicago, IL, USA). Associations between the methylation levels and various factors were analyzed using a Mann-Whitney *U* test. We divided all of the samples for the survival analysis into two classes using a lower tertiary cut-off point of 2.51 (log_10_ scale). The samples above this threshold were considered the high methylation group, and the samples below this point were considered the low methylation group. Survival curves were plotted using the Kaplan-Meier method, and differences in the probability curves between the low and high methylation groups were analyzed using the log-rank test (SPSS 12.0). The median periods of follow-up were 26.53 and 26.81 months for the low and high methylation groups, respectively.

We used multivariate Cox proportional hazards regression models to estimate the hazard ratios (HR) for the high methylation group compared to the low methylation group to further evaluate the significance of *LGALS4* methylation as a prognostic factor for the survival probability independent of covariates,. The covariates were included because of their significant associations with UC survival in a prior univariate analysis, with the exception of age and gender, which were forced into the model. These statistical analyses were performed using SAS win8e (SAS Institute, Cary, NC).

Bar charts were used to present the experimental data from the *in vitro* studies histographically. Comparisons of the phenotype changes between the transfectants with and without gal-4 expression were examined using Student's t test. Columns and bars represent the mean±SEM of three or four independent experiments performed in duplicate. Two-sided P values <0.05 were considered statistically significant.

## SUPPLEMENTARY DATA FIGURES AND TABLES





## Abbreviations

ACTB: β-actin gene
AJCC: American Joint Committee on Cancer
BSP: bisulfite sequencing polymerase chain reaction
CI: confidence interval
CRC: colorectal cancer
Δβ: beta difference
EMT: epithelial-to-mesenchymal transition
FFPE: formalin-fixed and paraffin-embedded
5-aza-dC: 5-aza-2′-deoxycytidine
gal-4: galectin-4
HR: hazard ratio
IHC: immunohistochemistry
LGALS4: lectin, galactoside-binding, soluble, 4
LN: lymph node
MS-PCR: methylation-specific polymerase chain reaction
NA: nonrecurrent-advanced-stage
NE: nonrecurrent-early-stage
nt: nucleotide
NTUH: National Taiwan University Hospital
qMSP: quantitative methylation-specific polymerase chain reaction
RA: recurrent-advanced-stage
RE: recurrent-early-stage
RT-PCR: reverse transcriptase-polymerase chain reaction
SEM: standard error of the mean
TSA: trichostatin A
TSGH-8301/gal-4: TSGH-8301 transfectants containing plasmid with gal-4
TSGH-8301/mock: TSGH-8301 transfectants containing plasmid without gal-4
T24/gal-4: T24 transfectants containing plasmid with gal-4
T24/mock: T24 transfectants containing plasmid without gal-4
UC: urothelial carcinoma.
