# Supplementary material for: Promoter hypermethylation of LGALS4 correlates with poor prognosis in patients with urothelial carcinoma
Source: Oncotarget. 2017 Mar 3;8(14):23787–802. doi: 10.18632/oncotarget.15865 (PMC5410344; doi:10.18632/oncotarget.15865)
Supplement: Supplementary file 1 [file oncotarget-08-23787-s001.pdf]

Promoter hypermethylation of *LGALS4* correlates with poor prognosis in patients with urothelial carcinoma

SUPPLEMENTARY DATA

DNA extraction

UC frozen tissues were lysed with 1.0 mL DNAzol reagent (Molecular Research Center, Cincinnati, OH, USA) and 200 µg/mL proteinase K (Sigma, St. Louis, MO, USA) by gently pipetting. A 0.5-mL aliquot of chloroform was added to the cell lysate. This mixture was shaken vigorously and then centrifuged at 12,000 rpm for 5 min. After centrifugation, the supernatant was collected and 1 mL 75% ethanol was added. The tube was agitated vigorously until the DNA pellet was completely dispersed. The resulting mixture was centrifuged at 12,000 rpm for 5 min at room temperature. Ethanol wash was decanted carefully and the tubes were stored vertically for approximately 1 min. The DNA pellet was dissolved in 50 µL 8 mM NaOH and incubated at room temperature for 3-5 min, followed by repetitive pipetting. DNA was quantified with NanoDrop ND-1000 spectrophotometer (Thermo Fisher Scientific, Waltham, MA, USA), with 1 µL of sample at 260 nm wavelength for determination of double-stranded DNA.

Immunohistochemistry

Protein expression of *LGALS4* in FFPE specimens was determined using an immunohistochemical assay based on the avidin-biotin-peroxidase method. Paraffin sections, 5-µm thick on microscope glass slides, were deparaffinized in xylene and rehydrated in graded ethanol and water. Endogenous peroxidase activity was then blocked by incubation with peroxidase blocking reagent containing 3% H_2O_2 (DAKO Corp, Carpinteria, CA, USA) according to the manufacturer's protocol. After heat-induced antigen retrieval, non-specific protein-binding sites and any sites with affinity for biotin were saturated. The sections were incubated with gal-4 primary antibody solution (Santa Cruz Biotechnology, Santa Cruz, CA, USA) at a final dilution of 1:100 for overnight at 4°C. The slides were washed and incubated with biotinylated secondary antibodies, and the bound markers were visualized using avidin–biotin–peroxidase complex kit reagents with 3, 3'-diaminobenzidine (DAB) in 0.03% H_2O_2 solution as the chromogenic substarte (DAKO). Slides were counterstained with hematoxylin, mounted, and coverslipped. Control sections were treated with PBS to exclude antigen-independent reactions.

RNA extraction and reverse transcriptase-PCR (RT-PCR)

Total RNA from cultured cells was extracted using TRI reagent (Molecular Research Center), according to the manufacturer's protocol. For RT-PCR analysis, total RNA was first incubated with RNase-free DNaseI at 37°C for 30 min to digest residual genomic DNA (Roche Diagnostics, Foster City, CA, USA), and 2 µg of resulting RNA was then reverse transcribed (RT) to synthesize cDNA for PCR amplification. RT reaction was carried out for 50 min at 50°C in a mixture of RT buffer (50 mM Tris/HCl, pH8.3, 75 mM KCl, 3 mM $MgCl_2$), 10 mM DTT, 10 mM each dNTPs, 0.5 µg random hexamer primers and 20 U SSRTII, and terminated by an incubation for 15 min at 70°C. Subsequently, PCR amplification was carried out in 20 µL reaction mixture, containing 2 µL cDNA, 0.5 U Tag polymerase, 50 mM KCl, 10 mM Tris/HCl (pH8.8), 1.5 mM $MgCl_2$, 50 µM each dNTPs, and 0.1 µM each paired primers. Nucleotide sequences for primer set were GCT CAA CGT GGG AAT GTC TGT (sense), and GAG CCC ACC TTG AAG TTG ATA (antisense). PCR condition was run as heating the samples to 94°C for 4 min, followed by 36 cycles of 94°C for 1 min, 60°C for 1 min, and 72°C for 2 min, and ending with an extension period of 10 min at 72°C. The PCR products were separated in 2% TAE (or 1% agarose) gels, stained by ethidium bromide, and visualized under UV light. Glyceraldehyde-3-phosphate dehydrogenase (GAPDH) was amplified and included in each gel electrophoresis run as an internal control.

5-aza-2'-deoxycytidine (5-aza-dC) and trichostatin A (TSA) treatment

We examined gene expression of *LGALS4* in several human urothelial cell lines exposed to 5-aza-dC (Sigma-Aldrich), a DNA methyltransferase inhibitor, and/or TSA (Sigma-Aldrich), a histone deacetylase inhibitor, to examine the potential involvement of epigenetic silencing in *LGALS4* repression. Cells were grown to a 25% confluency in 60-mm Petri dish on day 1. Cells were then cultured for another 72 h (day 2 ~ day 4) while replacing with fresh medium containing 1 µM 5-aza-dC or DMSO (as a negative control) every 24 h. TSA was added on day 3 at a concentration of 0.1 µM and cultured for 24 h. On day 4 after treatments, the cells were harvested for the extraction of RNA being used in subsequent RT-PCR analysis.

Western blotting analysis

Western blotting analysis was performed as described previously [1–2]. In brief, cells were harvested and lysed at 0°C in RIPA lysis buffer containing protease inhibitors (Thermo Fisher Scientific). After quantitation with the Bradford method (Bio-Rad Laboratory, Hercules, CA, USA), equal amounts of cell lysates were separated by SDS-PAGE on 10% polyacrylamide gel, and the proteins were transferred to PVDF membranes (GE Healthcare, Munich, Germany). After 1 h blocking, the membranes were washed and incubate overnight at 4°C with primary antibodies against gal-4 (Santa Cruz Biotechnology), and β-actin (Abcam, Cambridge, MA, USA) as a loading control. After incubation, secondary horseradish peroxidase-conjugated antibodies (Abcam) were used in PBST/1% BSA for an incubation of 60 min at room temperature. Bound antibodies were visualized using the SuperSignal West Pico Chemiluminescent Substrate (Pierce Biotechnology, Rockford, IL, USA).

REFERENCES

1. Su PF, Hu YJ, Ho IC, Cheng YM, Lee TC. Distinct gene expression profiles in immortalized human urothelial cells exposed to inorganic arsenite and its methylated trivalent metabolites. Environ Health Perspect. 2006; 114:394-403.
2. Wang HH, Wu MM, Chan MW, Pu YS, Chen CJ, Lee TC. Long-term low-dose exposure of human urothelial cells to sodium arsenite activates lipocalin-2 via promoter hypomethylation. Arch Toxicol. 2014; 88:1549-59.

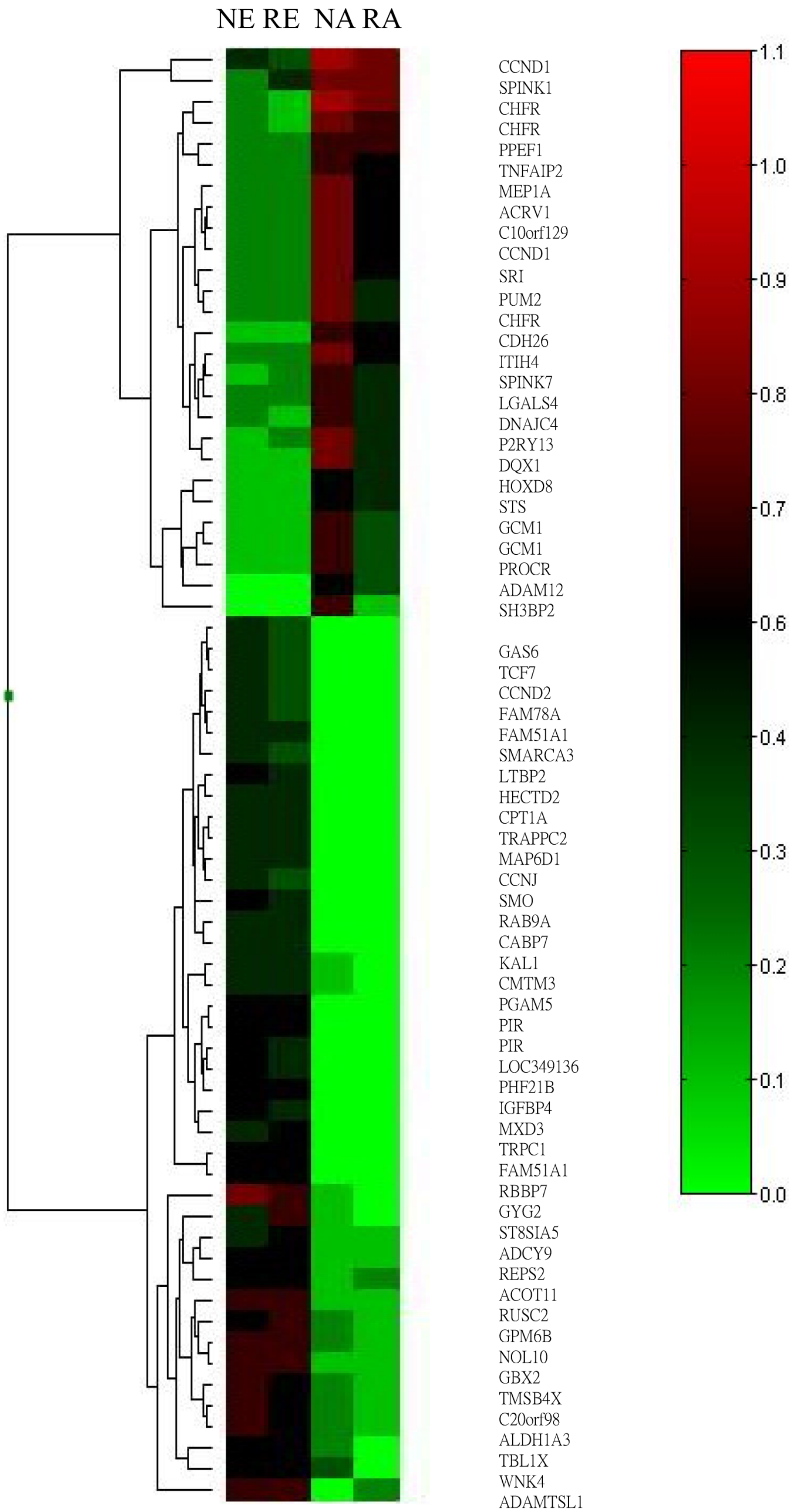

Supplementary Figure 1: Heatmap of the methylation profiles showing 69 CpG sites with absolute beta difference greater than 0.4 for early (NE or RE)- and advanced (NA or RA)-stage UC groups. The heatmap shows the intensity of methylation with 0 (green) indicating unmethylated and 1 (red) indicating fully methylated. The columns represent UC groups, and the rows represent the 69 CpG sites. Tumor DNA samples were obtained from NE, RE, NA, and RA groups with various stages of UC progression. Each group consisting of 4 patient samples for pooled DNA. NE: nonrecurrent-early-stage. RE: recurrent-early-stage. NA: nonrecurrent-advanced-stage. RA: recurrent-advanced-stage.

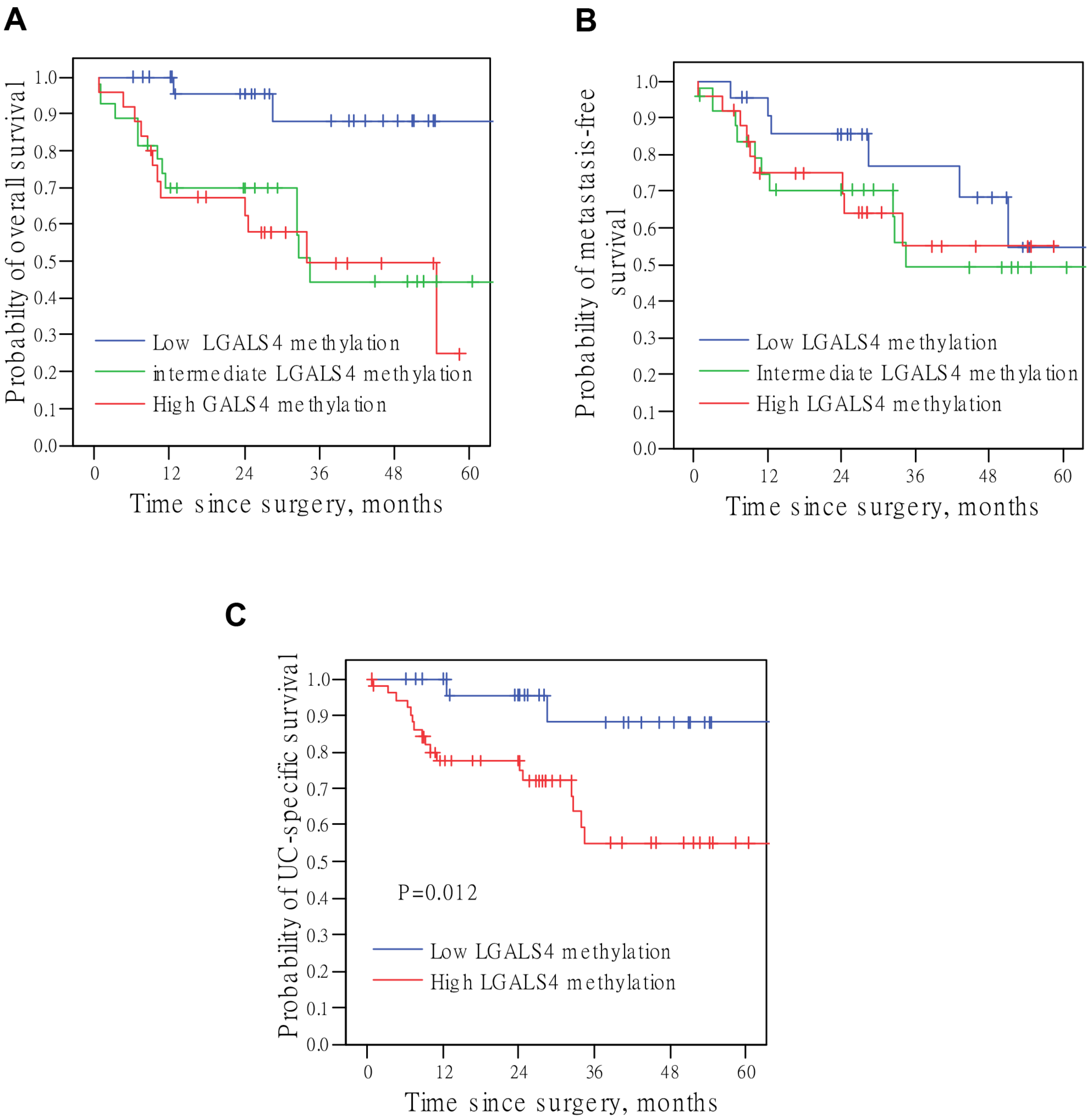

Supplementary Figure 2: Kaplan-Meier curves for survival analysis with log-rank P values. (A) Overall survival probability. **(B)** Metastasis-free survival probability. *LGALS4* methylation level of 2.51 and 3.40 in $\log_{10}$ scales were the cut-off points to classify samples into low, intermediate, or high methylation group. These values approximately represent the respective lower and upper tertiary values of the distribution in all patient subjects. **(C)** Urothelial carcinoma (UC)-specific survival probability. *LGALS4* methylation level of 2.51 in a $\log_{10}$ scale was the cut-off point to classify samples into low or high methylation group.

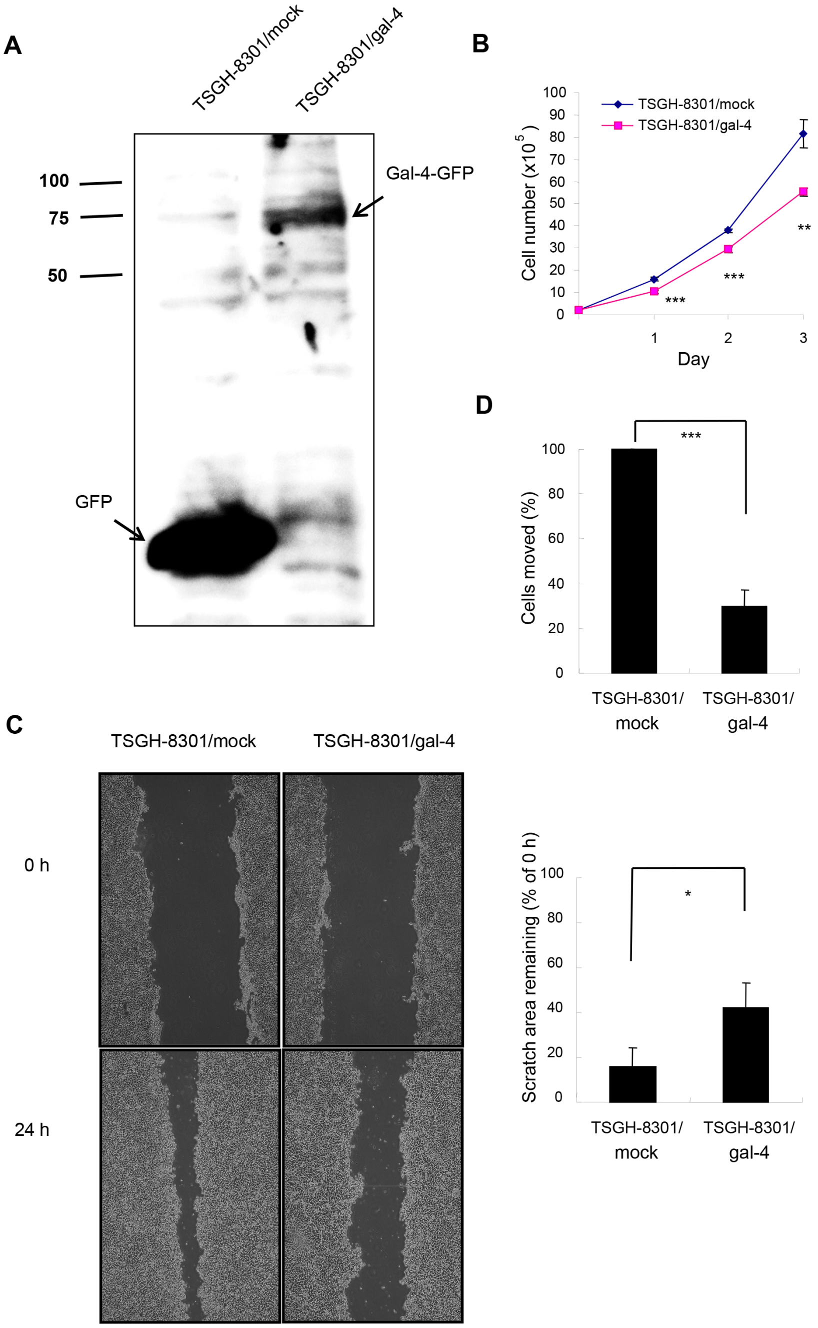

Supplementary Figure 3: Effects of *LGALS4* protein expression (gal-4) in TSGH-8301 transfectants. The cells were transfected with either the pCMV6-AC-GFP/gal-4 (TSGH-8301/gal-4 cell line) or empty vector (TSGH-8301/mock cell line) as a control. **(A)** Ectopic expression of gal-4 in TSGH-8301 transfectants. Proteins from the whole extracts of TSGH-8301/mock and TSGH-8301/gal-4 cells were analyzed by Western analysis for the detection of GFP only and Gal-4-GFP, respectively. **(B)** Cell proliferation of TSGH-8301/mock and TSGH-8301/gal-4 cells, as determined in cell viability assays using trypan blue exclusion method. Bars represent the mean± SEM of three independent experiments performed in duplicate. ** P<0.01, and *** P<0.001. **(C)** Migration capability of TSGH-8301/mock and TSGH-8301/gal-4, as measured in wound healing scratch assay. Representative photographs at time points 0 and 24 h after a scratch treatment. Histographic presentation of the data analyzed from the photographs taken at 0 h and 24 h after the scratch. Columns and bars represent the mean± SEM derived from three separate experiments. *** P<0.001. **(D)** Invasion capability of TSGH-8301/mock and TSGH-8301/gal-4, as assessed by moving over gelatin-coated transwell chambers. Columns and bars represent the mean±SEM derived from three independent experiments. * P<0.05.

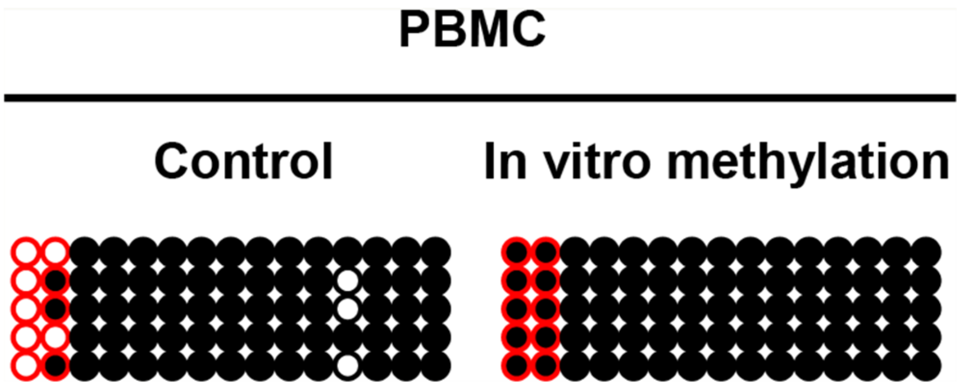

Supplementary Figure 4: Results of bisulfite sequencing PCR (BSP) for the *in vitro* methylated leukocyte DNA. Each row illustrates a clone, and column a single CpG site. Open and filled circles represent methylated and unmethylated CpG, respectively. A total of 15 CpG sites within the promoter of *LGALS4* spanning -252 to +184 nt. were determined. The first two CpG dinucleotides were analyzed in qMSP assay including the significant loci -194 nt. identified from analysis results by the Infinium Methylation 27K BeadChip assay. PBMC: peripheral blood mononuclear cells.

Supplementary Table 1: TNM category and overall cancer stage* of 16 patient samples for DNA methylation profiling

	Non- recurrent								Recurrent							
	Early stage				Advanced stage				Early stage				Advanced stage			
Patient ID	#18	#22	#30	#34	#1	#62	#70	#72	#11	#15	#21	#33	#4	#57	#68	#77
Age	53	72	52	68	66	77	55	49	79	62	59	52	63	68	59	71
Gender	M	F	M	F	M	F	M	F	M	M	F	F	M	M	F	F
Location of tumor**	1	1	3	1	2	1	1	1	1	1	1	1	3	1	1	1
Histological grade	2	2	L	2	H	H	H	H	L	2	2	2	3	3	H	H
Primary tumor (T)	1a	1	a	2	3	3	3	3	1a	1a	1	2	3	4a	3	3
Regional Lymph nodes (N)	0	0	0	0	0	0	0	0	0	0	0	0	0	0	2	0
Distant metastasis (M)	0	0	0	0	0	0	0	0	0	0	0	0	0	0	0	0
Overall cancer stage	I	I	0a	II	III	III	III	III	I	I	I	II	III	III	IV	III

* The American Joint Committee on Cancer (AJCC) TNM system was used. Individual T, N, and M categories were combined to find the overall cancer stage.

** 1: the urinary bladder, 2: the ureter, 3: the renal pelvis.

Supplementary Table 2: Genes differentially (|Δβ| ≥ 0.4)* methylated between early- and advanced-stage urothelial carcinoma

See Supplementary File 1
