# Supplementary material for: Promoter hypermethylation of LGALS4 correlates with poor prognosis in patients with urothelial carcinoma
Source: Oncotarget. 2017 Mar 3;8(14):23787–802. doi: 10.18632/oncotarget.15865 (PMC5410344; doi:10.18632/oncotarget.15865)
Supplement: Supplementary file 2 [file oncotarget-08-23787-s002.doc]

**Supplementary Table 2: Genes differentially (Δβ ≥ 0.4)^*^ methylated between early- and advanced-**stage urothelial carcinoma

| Accession number | Gene symbol | Gene annotation | Delta beta |
| --- | --- | --- | --- |
| Hypermethylated genes | | | |
| NM_020069.3 | ACRV1 | acrosomal vesicle protein 1 | 0.4128 |
| NM_003474.3 | ADAM12 | ADAM metallopeptidase domain 12 | 0.4094 |
| NM_207321.1 | C10orf129 | chromosome 10 open reading frame 129 | 0.4322 |
| NT_078088.3 | CCND1 | cyclin D1 | 0.4489 |
| NT_078088.3 | CCND1 | cyclin D1 | 0.4367 |
| NM_177980.1 | CDH26 | cadherin 26 | 0.4422 |
| NT_024477.13 | CHFR | checkpoint with forkhead and ring finger domains | 0.6207 |
| NT_024477.13 | CHFR | checkpoint with forkhead and ring finger domains | 0.5308 |
| NT_024477.13 | CHFR | checkpoint with forkhead and ring finger domains | 0.4009 |
| NM_005528.1 | DNAJC4 | DnaJ (Hsp40) homolog, subfamily C, member 4 | 0.4193 |
| NM_133637.1 | DQX1 | RNA-dependent ATPase homolog with a novel DEAQ box | 0.4322 |
| NM_003643.2 | GCM1 | glial cells missing homolog 1 | 0.4176 |
| NM_003643.2 | GCM1 | glial cells missing homolog 1 | 0.4125 |
| NM_019558.2 | HOXD8 | homeobox D8 | 0.4098 |
| NM_002218.3 | ITIH4 | inter-alpha (globulin) inhibitor H4 | 0.4268 |
| NM_006149.2 | LGALS4 | lectin, galactoside-binding, soluble, 4 | 0.4115 |
| NM_005588.1 | MEP1A | meprin A, alpha (PABA peptide hydrolase) | 0.4119 |
| NM_023914.2 | P2RY13 | purinergic receptor P2Y, G-protein coupled, 13 | 0.4120 |
| NM_152223.1 | PPEF1 | protein phosphatase, EF-hand calcium binding domain 1 | 0.4510 |
| NM_006404.3 | PROCR | protein C receptor, endothelial | 0.4003 |
| NM_015317.1 | PUM2 | pumilio homolog 2 | 0.4160 |
| NM_003023.2 | SH3BP2 | SH3-domain binding protein 2 | 0.4027 |
| NM_003122.2 | SPINK1 | serine peptidase inhibitor, Kazal type 1 | 0.4507 |
| NM_032566.2 | SPINK7 | serine-type endopeptidase inhibitor activity | 0.4102 |
| NM_198901.1 | SRI | sorcin | 0.4737 |
| NM_000351.3 | STS | steroid sulfatase (microsomal), isozyme S | 0.4066 |
| NM_006291.2 | TNFAIP2 | tumor necrosis factor, alpha-induced protein 2 | 0.4367 |
| Hypomethylated genes | | | |
| NM_015547.2 | ACOT11 | acyl-CoA thioesterase 11 | -0.4079 |
| NM_052866.2 | ADAMTSL1 | ADAMTS-like 1 | -0.4920 |
| NM_001116.2 | ADCY9 | adenylate cyclase 9 | -0.4163 |
| NM_000693.1 | ALDH1A3 | aldehyde dehydrogenase 1 family, member A3 | -0.4475 |
| NM_024958.1 | C20orf98 | neurensin 2 | -0.4184 |
| NM_182527.1 | CABP7 | calcium binding protein 7 | -0.4267 |
| NM_001759.2 | CCND2 | cyclin D2 | -0.4014 |
| NM_019084.2 | CCNJ | cyclin J | -0.4676 |
| NM_144601.2 | CMTM3 | CKLF-like MARVEL transmembrane domain containing 3 | -0.4070 |
| NM_001031847.1 | CPT1A | carnitine palmitoyltransferase 1A | -0.5141 |
| NM_017856.1 | FAM51A1 | Gemin8 | -0.6108 |
| NM_017856.1 | FAM51A1 | Gemin8 | -0.4289 |
| NM_033387.2 | FAM78A |  | -0.4195 |
| NM_000820.1 | GAS6 | growth arrest-specific 6 | -0.4064 |
| NM_001485.2 | GBX2 | gastrulation brain homeobox 2 | -0.4913 |
| NM_001001995.1 | GPM6B | glycoprotein M6B | -0.4501 |
| NM_003918.1 | GYG2 | glycogenin 2 | -0.6456 |
| NM_173497.1 | HECTD2 | HECT domain containing 2 | -0.5335 |
| NM_001552.2 | IGFBP4 | insulin-like growth factor binding protein 4 | -0.5358 |
| NM_000216.1 | KAL1 | Kallmann syndrome 1 sequence | -0.4243 |
| NM_198285.1 | LOC349136 |  | -0.4904 |
| NM_000428.2 | LTBP2 | latent transforming growth factor beta binding protein 2 | -0.4060 |
| NM_024871.1 | MAP6D1 | microtubule-associated protein 6 isoform 1 | -0.4828 |
| NM_031300.2 | MXD3 | MAX dimerization protein 3 | -0.4439 |
| NM_024894.1 | NOL10 | nucleolar protein 10 | -0.4733 |
| NM_138575.1 | PGAM5 | phosphoglycerate mutase family member 5 | -0.4374 |
| NM_138415.1 | PHF21B | PHD finger protein 21B | -0.5069 |
| NM_003662.2 | PIR | Pirin | -0.5205 |
| NM_003662.2 | PIR | Pirin | -0.5153 |
| NM_004251.3 | RAB9A | RAB9A, member RAS oncogene family | -0.4373 |
| NM_002893.2 | RBBP7 | retinoblastoma binding protein 7 | -0.6051 |
| NM_004726.1 | REPS2 | RalBP1-associated Eps domain-containing protein 2 | -0.4326 |
| NM_014806.1 | RUSC2 | RUN and SH3 domain containing 2 | -0.5298 |
| NM_003071.2 | SMARCA3 | helicase-like transcription factor | -0.4506 |
| NM_005631.3 | SMO | smoothened homolog | -0.4403 |
| NM_013305.3 | ST8SIA5 | ST8 alpha-N-acetyl-neuraminide alpha-2,8-sialyltransferase 5 | -0.4963 |
| NM_005647.2 | TBL1X | transducin (beta)-like 1X-linked | -0.4854 |
| NM_201633.1 | TCF7 | transcription factor 7 | -0.4003 |
| NM_021109.2 | TMSB4X | thymosin beta 4, X-linked | -0.4585 |
| NM_014563.3 | TRAPPC2 | trafficking protein particle complex 2 | -0.4732 |
| NM_003304.3 | TRPC1 | transient receptor potential cation channel, subfamily C, member 1 | -0.4510 |
| NM_032387.3 | WNK4 | WNK lysine deficient protein kinase 4 | -0.4242 |

^*^Δβ : absolute beta difference value, the absolute difference of methylation beta value between two sample groups detected using the Infinium Methylation 27K BeadChip assay.
